# Dengue Hemorrhagic Fever in Quang Nam Province (Vietnam) from 2020 to 2022—A Study on Serotypes Distribution and Immunology Factors

**DOI:** 10.3390/diagnostics14161772

**Published:** 2024-08-14

**Authors:** Huong T. Pham, Thao N. T. Pham, Nhu H. T. Tran, Quang D. Ha, Duy K. Tran, Nam H. D. Nguyen, Van H. Pham, Son T. Pham

**Affiliations:** 1 Vietnam Research and Development Institute of Clinical Microbiology, Ho Chi Minh City 700000, Vietnam; phamthienhuong@gmail.com (H.T.P.); hongnhutran9595@gmail.com (N.H.T.T.); haquyquang2601@gmail.com (Q.D.H.); khanhduytransh@gmail.com (D.K.T.); 2 Nam Khoa Co., Ltd., Ho Chi Minh City 700000, Vietnam; 3 Faculty of Medicine, Phan Chau Trinh University, Dien Ban 520000, Vietnam; thao.ptn.yk1@pctu.edu.vn (T.N.T.P.); nam.ndh@pctu.edu.vn (N.H.D.N.); 4 New South Wales Health, Sydney, NSW 2065, Australia; 5 Royal Australian College of General Practitioners, Sydney, NSW 2000, Australia; 6 Australasian College for Emergency Medicine, Melbourne, VIC 3003, Australia; 7 Sydney Medical School, The University of Sydney, Sydney, NSW 2145, Australia

**Keywords:** Dengue virus, serotypes, MLP RT-rPCR, secondary infection

## Abstract

*Background:* Dengue hemorrhagic fever (DHF) is the most prevalent and fastest-growing vector-borne disease globally, with symptoms ranging from mild to severe and, in some cases, fatal. Quang Nam province in Vietnam can serve as a model for dengue epidemiological study, as it is an endemic region for DHF with a tropical climate, which significantly constrains the health system. However, there are very few epidemiological and microbiological reports on Dengue virus (DENV) serotypes in this region due to the limited availability of advanced surveillance infrastructure. *Aims of the study:* This study aims to (1) assess the PCR positivity rates among hospitalized patients with clinical Dengue presentation; (2) identify the circulating DENV serotypes; and (3) assess the impact of secondary DENV infections on outbreak severity by detecting the presence of DENV-specific IgG antibodies in the plasma of DENV-infected patients. *Materials and methods:* Blood samples from patients clinically diagnosed with DHF and admitted to Quang Nam General Hospital (2020–2022) were analyzed. RNA extraction was performed using the ^NK^DNA/RNAprep MAGBEAD kit, followed by Multiplex Reverse Transcription real-time Polymerase Chain Reaction (MLP RT-rPCR) for DENV detection and serotype identification. Positive samples were further tested for DENV-specific IgG antibodies using an enzyme-linked immunosorbent assay (ELISA). *Results:* The PCR positivity rate among hospitalized patients was approximately 68% throughout the study period. A significant shift in DENV serotypes was observed, with DENV-2 initially dominant and later giving way to DENV-1. IgG was detected in nearly half of the MPL RT-rPCR-positive samples, indicating secondary DENV infections. *Conclusions:* Our study highlights persistent dengue prevalence and dynamic shifts in DENV serotypes in Quang Nam province, emphasizing the need for improved diagnostic strategies and timely sample collection. The significant serotype shifts and the presence of IgG in hospitalized patients suggest potential severe outcomes from recurrent DENV infections, possibly linked to antibody-dependent enhancement (ADE) effect, underscoring the importance of advanced surveillance, vector control, vaccination campaigns, and public education to predict and prevent future DHF epidemics.

## 1. Introduction

Dengue virus (DENV) is an RNA virus of the Flaviviridae family. It is the main cause of Dengue hemorrhagic fever (DHF), the most prevalent anthropod-borne viral illness in humans, and is transmitted by the *Aedes aegypti* (*A. aegypti*) mosquito. Clinical manifestations of DHF vary from mild or no symptoms to severe and even death [1]. According to reports of the World Health Organization (WHO), cases of DHF have increased dramatically from 505,430 cases in 2000 to 5.2 million in 2019 [2]. DHF is currently endemic in over 100 countries in different regions of the WHO, especially those in Africa, the Americas, the Eastern Mediterranean, South East Asia, and the Western Pacific. The highest number of cases was recorded in 2023, with a historic high of over 6.5 million cases and more than 7300 dengue-related deaths. Risk factors of infection include tropical and subtropical areas, in combination with unplanned urbanization and lack of population’s knowledge [2].

Four different serotypes of DENV, from DENV-1 to DENV-4, have been discovered globally [1]. Different serotypes could influence the manifestations and severity of DHF [3,4]. Secondary DENV infection with a DENV serotype distinct from the primary serotype is the greatest risk factor for dengue hemorrhagic fever/dengue shock syndrome (DHF/DSS). This may be due to the antibody-dependent enhancement (ADE) effect [5,6,7,8].

In Vietnam, infections occur year-round with all four serotypes responsible [9]. According to the WHO, from 2004 to 2010, Vietnam ranked third in the number of average incidence of DHF each year [10]. In Quang Nam, a province in the central part of Vietnam, there were consistently over 100 cases per 100,000 people from 2015 to 2020. In 2022, the Health Office and CDC of Quang Nam province reported the highest number of DHF cases in the Central region of Vietnam, reaching 21,306 cases, with one recorded death [11].

The diverse clinical manifestations and severity, as well as the risk of severe outcomes in different DENV serotypes, highlight the importance of understanding the distribution of these serotypes for epidemiological assessment. Most studies conducted in Vietnam focused on the northern and southern regions [12,13,14], whereas there are few reports in the central part due to a lack of laboratory facilities. This makes the transmission dynamics relevant environmental risk factors, and the distribution of DENV serotypes in this region is not fully understood.

## 2. Aims of the Study

We aimed to provide valuable insights into the epidemiology of DENV in Quang Nam province from 2020 to 2022 by following the main objectives: (1) assess the PCR positivity rates among hospitalized patients with clinical Dengue presentation; (2) identify the circulating DENV serotypes; and (3) assess the impact of secondary DENV infections on outbreak severity by detecting the presence of DENV-specific IgG antibodies in the plasma of DENV-infected patients

## 3. Materials and Methods

### 3.1. Subjects and Sample Collection

This was a cross-sectional retrospective study. A total of 1167 EDTA blood samples were collected on the first day of hospitalization from all patients who were hospitalized in Quang Nam General Hospital with the clinical diagnosis of DHF following the WHO’s criteria between 2020 and 2022 [15]. There were no exclusion criteria. After collection, the samples were stored at 2 °C to 8 °C and transported to the Vietnam Research and Development Institute of Clinical Microbiology laboratory in Ho Chi Minh City within 72 h. During transportation, the samples were packaged in foam boxes with gel ice packs. Each sample was labeled with the patient’s age, gender, and date of collection.

### 3.2. Nucleic Acid Extraction

All samples were centrifuged at 3000 RPM for 10 min to collect the plasma. Subsequently, nucleic acids were extracted from the plasma using ^NK^DNARNAprepMAGBEAD kits produced by Nam Khoa Biotek Co., Ltd. (Ho Chi Minh City, Vietnam), following the manufacturer’s instructions, and stored at −70 °C [16,17].

### 3.3. One-Step RT rPCR for the Detection of DENV Serotypes

The extraction product (5 μL) was added to 15 μL of one-step RT rPCR mix containing four specific primer pairs and four specific probes for four DENV serotypes. The sequences of the primers and probes, as shown in Table 1, were referred from Gilberto A. Santiago (CDC) et al. [18,19]. These primers and probes were ordered from Proligo (Sigma, Singapore).

The multiplex Reverse Transcription real-time Polymerase Chain Reaction (MLP RT-rPCR) was performed on CFX96 instrument (Bio-rad, Hercules, CA, USA) with the following thermal cycling protocol: 45 °C for 10 min for reverse transcription (RT), 95 °C for 10 min to destroy the RT enzyme and activate the hot-start taq polymerase, 40 cycles of two thermal steps at 95 °C for 15 s, followed by 60 °C for 1 min. The results were read to determine the positivity and DENV serotypes based on the amplifying signals with four different color channels: FAM for DENV 1, HEX for DENV 2, TexasRED for DENV 3, and CY5 for DENV 4. If no signals were detected in all channels, we proceeded with an additional assay utilizing an RT-rPCR mix produced by Nam Khoa Biotek Co., Ltd. (Ho Chi Minh City, Vietnam) targeting the house-keeping gene, RNAseP, to verify the negative status of the sample.

### 3.4. Detection of DENV-Specific Antibodies

Positive samples identified by MPL RT-rPCR were selected for the detection of IgG DENV-specific antibodies. This was performed using the enzyme-linked immunosorbent assay (ELISA) of the SERION ELISA classic Dengue Virus IgG kit (Serion GmbH, Wurzburg, Germany) following the manufacturer’s manual.

### 3.5. Analysis of the Results

For statistical analysis, we used SPSS 25.0 statistical software and the chi-square test for the correlation of different factors (*p* < 0.05 was considered statistically significant).

## 4. Results

### 4.1. Among All Hospitalized Patients with Dengue Clinical Diagnosis, 68.64% Were Positive on PCR Detected by MPL RT-rPCR

We collected a total of 1167 samples from patients clinically diagnosed with dengue infections and hospitalized during the study period between 2020 and 2022: 122 samples in 2020–2021 and 1045 samples in 2022. Overall, 801 samples were tested positive for DENV using MPL RT-rPCR, resulting in a PCR positivity rate of 68.64%. This rate indicated that not all patients admitted with a clinical diagnosis of DHF had detectable levels of the virus using PCR testing. A detailed breakdown of positivity rates by year is presented in Table 2.

The PCR positivity rates were fairly consistent between the two periods, with a slight decrease in positivity rate from 68.85% in 2020–2021 to 68.61% in 2022, despite the significantly lower number of samples collected in 2020–2021 compared to 2022. This stability in PCR positivity rates suggests that the prevalence of DENV remained relatively unchanged over the study period, indicating a persistent burden of dengue in the region. The consistent detection rates reflect the endemic nature of dengue in Quang Nam province, likely influenced by factors such as vector population dynamics and environmental conditions conducive to mosquito breeding.

### 4.2. The Distribution of Dengue Infection May Not Be Age- or Gender-Specific

The study included 621 female and 546 male participants. The PCR positivity rates were slightly higher in females (51.69%) compared to males (48.31%), though this difference was not statistically significant (*p* = 0.121). The positivity rate was 66.67% for females and 70.88% for males. However, this difference was not statistically significant (*p* > 0.05), as detailed in Table 3. These results do not indicate a substantial gender-based difference in DENV infection rates.

Regarding age distribution, the samples were categorized into two groups—younger than 16 years old and older than 16 years old. Among the 801 PCR-positive samples, individuals ≥16 years old accounted for the majority of 784 samples (97.88%), while only 17 samples (2.12%) were from those < 16 years old. Noteworthy, the positivity rates were 68.89% (17/29) and 58.62% (784/1138) for each group, respectively. This age difference was also not statistically significant (*p* > 0.05), as shown in Table 4. However, the low representation of the younger age group may limit the ability to draw statistically significant conclusions about the differences in positivity rates. Although the age difference was not statistically significant, the predominance of cases in older individuals suggests a shift in the demographic profile of DHF patients, possibly due to changes in population structure and urbanization patterns.

### 4.3. The Epidemiological Distribution of DENV Serotypes Revealed Significant Dynamic Shifts over Time

**Figure 1:**
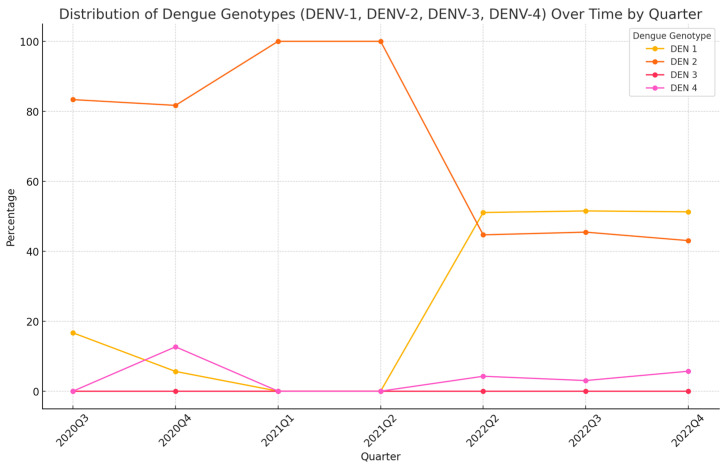
**Distribution of dengue serotypes (DENV-1, DENV-2, DENV-3, DENV-4) over time**. This figure shows the percentage distribution of Dengue virus genotypes DENV-1, DENV-2, DENV-3, and DENV-4 in Quang Nam province, Vietnam, from 2020 to 2022 in each annual quarter. The x-axis represents the quarters of each year (Q1 = January–March; Q2 = April–June; Q3 = July–September; Q4 = October–December), while the y-axis indicates the percentage of each Dengue serotype. The data points are connected by lines to illustrate the trends over time. Notably, DENV-2 was predominant in 2020–2021, whereas DENV-1 became the most prevalent in 2022. DENV-3 was not detected in any of the samples throughout the study period. The fluctuations in genotype distributions underscore the dynamic nature of Dengue epidemiology in the region.

During the study period, the distribution of DENV serotypes varied significantly between 2020 and 2021 and 2022. In 2020–2021, DENV-2 was the predominant serotype, found in 83.33% of the positive samples, while DENV-1 and DENV-4 accounted for 5.95% and 10.71%, respectively. In 2022, DENV-1 became the most prevalent serotype, reaching 51.32%, followed by DENV-2 at 43.24% and DENV-4 at 5.44%. No DENV-3 cases were detected throughout the entire study period. Detailed percentages and numbers are shown in Table 5, and the distribution of DENV serotypes over time is demonstrated in Figure 1. This shift in serotype dominance is epidemiologically significant, as it can affect the clinical severity of infections and influence future outbreak risks. The emergence of DENV-1 as the dominant serotype in 2022 may indicate a potential change in the viral landscape and immunity patterns in the population, necessitating continuous monitoring and adaptation of public health strategies.

The overall change in serotype distribution between the two periods was statistically significant (*p* < 0.001), indicating a notable shift in the prevalence of DENV serotypes over time.

### 4.4. Secondary DENV Infection Occurred in Nearly Half of PCR-Positive Cases

**Figure 2:**
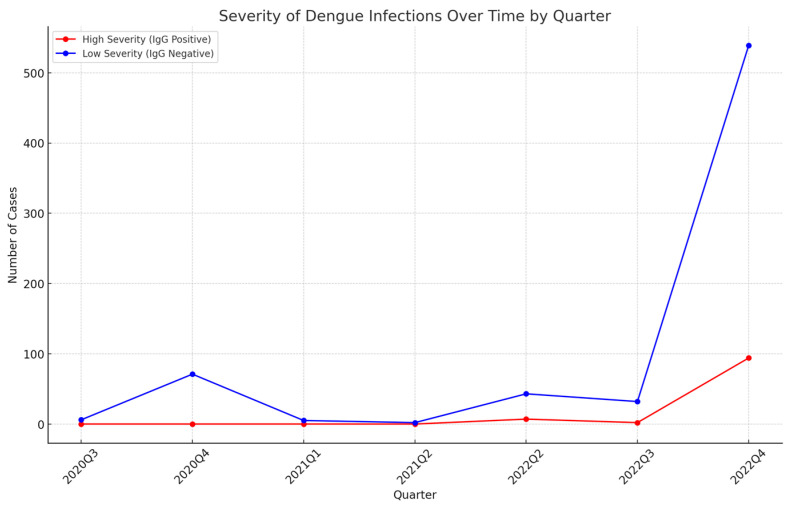
**Severity of dengue infections over time by quarter**. This figure illustrates the severity of Dengue infections in Quang Nam province, Vietnam, from 2020 to 2022, categorized by IgG positivity as a proxy for severity. The x-axis represents the quarters of each year, while the y-axis indicates the number of cases. The red line represents the number of high-severity cases (IgG-positive), and the green line represents the number of low-severity cases (IgG-negative). The trends highlight the fluctuations in the severity of Dengue infections over time, emphasizing the variation in the number of severe cases across different quarters. Although low-severity cases dominate most of the data, there is a noticeable increasing trend in high-severity cases over time, which raises concerns about the growing severity of Dengue infections in the region.

We collected all of the 801 positive serum samples for ELISA testing to detect the IgG antibody specific for DENV. The results revealed that IgG antibody was present in 385 samples (48.06%), indicating that the DHF patients associated with these samples were infected with a DENV serotype different from that of their previous DENV infection (Figure 2). The presence of IgG indicates past exposure to DENV and implies that these secondary infections could potentially lead to more severe disease outcomes, such as Dengue hemorrhagic fever (DHF) or Dengue shock syndrome (DSS), due to the antibody-dependent enhancement (ADE) effect. The trend in the increasing severity of dengue cases over time, as observed in the data, underscores the need for enhanced surveillance and public health interventions to mitigate the impact of secondary infections.

## 5. Discussion

### 5.1. Epidemiological Distribution of DENV between 2020 and 2022

Vietnam, especially the central region, including Quang Nam province, remains an endemic area for dengue hemorrhagic fever (DHF) due to high *A. aegypti* density, favorable climatic conditions, and population characteristics [20,21]. This study addressed three main objectives: (1) to assess the PCR positivity rates among hospitalized patients from dengue clinical diagnoses; (2) to identify the circulating DENV serotypes; and (3) to evaluate the impact of secondary DENV infections on outbreak severity by detecting the presence of DENV-specific IgG antibodies in the plasma of DENV-infected patients.

The overall PCR positivity rate reported in this study was 68.64% among all hospitalized patients who were diagnosed with Dengue from clinical presentations from 2020 to 2022. Several factors could contribute to this lower PCR positivity rate. One major factor is the timing of sample collection. PCR testing is most effective during the acute phase of the infection when viral RNA is present in the bloodstream. If samples are collected too late, the viral load may have decreased below detectable levels [22,23]. Additionally, patients in later stages of the disease may have already begun to seroconvert, producing antibodies that clear the virus from the bloodstream [24,25]. Another factor is the viral load itself. Some patients may have a low viral load that falls below the sensitivity threshold of the PCR test. This can occur naturally in the progression of the disease [25]. Furthermore, clinical diagnosis of dengue is based on symptoms that overlap with other febrile illnesses, such as other viral infections, leptospirosis, malaria, or bacterial infections [2,10,26]. Misdiagnosis can lead to the inclusion of patients without dengue in the study. Dengue symptoms can be non-specific and may resemble other viral or bacterial infections, resulting in a clinical diagnosis without PCR confirmation. Sample handling and storage also play a critical role. Improper handling, transportation, or storage of blood samples can lead to RNA degradation, affecting the PCR results. Issues with sample contamination or the quality of reagents can also impact PCR test accuracy. Nevertheless, when broken down by year, a slight decrease from 68.85% in 2020–2021 to 68.61% in 2022 was observed, suggesting relative stability in the detection rates of DENV infection among hospitalized patients with clinical diagnosis of DHF over the years. This aligned with research carried out in 2018 by Phan, D.Q. et al. on the same population which reported a positivity rate of 68.85% [21]. The stability in Dengue prevalence is likely due to unchanged environmental factors and public health measures, while the uniform diagnostic methodology ensured reliable detection across all samples. Together, these factors confirm that the observed rates accurately reflect the true prevalence of DENV infections in the region, which did not exhibit significant year-to-year fluctuations, reflecting a persistent burden of the disease in the studied population.

### 5.2. Identification of Circulating DENV Serotypes

All four DENV serotypes have been reported in Vietnam, each dominating during different periods. During our study period, the distribution of DENV serotypes varied significantly between 2020 and 2021 and 2022 (Table 5, Figure 1). In 2020–2021, DENV-2 was the predominant serotype found in 83.33% of the positive samples, while DENV-1 and DENV-4 accounted for 5.95% and 10.71%, respectively. In 2022, DENV-1 became the most prevalent serotype, reaching 51.32%, followed by DENV-2 at 43.24% and DENV-4 at 5.44%. No DENV-3 cases were detected throughout the study period. This shift in DENV serotype distribution could have significant epidemiological implications. The emergence of DENV-1 as the dominant serotype in 2022, replacing DENV-2, suggests a dynamic change in the viral landscape. Such shifts are critical to monitor because they can influence the severity and spread of future outbreaks. The presence of multiple serotypes in a region can also complicate vaccine strategies and increase the risk of secondary infections, which can lead to more severe disease outcomes due to antibody-dependent enhancement (ADE). According to the National Institute of Hygiene and Epidemiology, DENV-1 and DENV-2 are the dominant serotypes and circulate almost every year. DENV-3 emerged in the late 1990s and was the predominant serotype during the outbreak in 1998, while DENV-4 was detected between 1999 and 2003 [27]. The WHO suggests that the emergence of previously less common serotypes may indicate an increased risk of an outbreak for that year or the following year [28]. This trend is evident in recent studies from Vietnam. In 2016, a study in Southern Vietnam reported DENV-4 as the predominant serotype at 40.7%, while other serotypes contributed smaller shares [29]. By 2018, Phan, D.Q et al. also found that DENV-4 remained dominant at 68.5%, while DENV-2, DENV-1, and DENV-3 accounted for 17.95%, 12.83%, and 0.37% of the cases, respectively [21]. The dynamic shifts of DENV serotype reported in this study align with the WHO’s observations about the potential for outbreak risks associated with changes in serotype prevalence.

### 5.3. Age and Gender Distribution

Several gender-based studies on the prevalence of DENV infection have been conducted globally. Some reports suggest that the incidence is higher in males than in females, while others indicate similar rates between genders. Certain studies in India, such as by Prakash Om et al. (2015) and the Microbiology Department of Uttar Pradesh University of Health Sciences (2020), also found that the incidence in males was higher than in females, with male-to-female ratios of 1.78/1 and 1.54/1, respectively [30,31]. In our study, the reported percentages of PCR-positive males and female are 48.31% and 51.69%, respectively (male/female = 1/1.07). This minor variation might reflect differences in exposure risk, health-seeking behavior, or immune responses between genders within the region. Notably, these factors did not create a statistically significant difference in positivity rates (*p* = 0.121), indicating no substantial gender-based disparity in DENV infection rates within the studied population.

DENV infection can affect all age groups. Before the 2000s, the incidence of DHF was primarily concentrated in those under 15 years old. Specifically, between 1978 and 1992, the majority of DHF cases occurred in children under 9 years old [32]. Similarly, in 1998 and 1999, 90% of the cases were reported in children under 15 [33]. Many recent studies indicate that dengue fever is no longer confined to children but has shifted towards adulthood, with most cases now occurring in patients aged 20 and older [34,35]. Our study recorded the distribution of PCR-positive cases by age as 2.12% for those under 16 and predominantly 97.88% for those aged 16 and older, with positivity rates of 58.62% and 68.89%, respectively. Although the difference was not statistically significant, this aligned with the age structure reported by the General Statistics Office in 2022, with the majority of Vietnam’s population in the working age group (15–64 years) making up 67.4%, while those under 15 and those 65 and older constitute 24.1% and 8.5%, respectively [36]. 

Moreover, while the absolute number of cases under 16 is small compared to the older group (29 cases versus 1138 cases), the PCR positivity rate among younger patients is noteworthy and should not be considered negligible. This rate suggests that although fewer children presented with clinical symptoms severe enough to warrant hospitalization, those who did were significantly affected, with more than half testing positive for DENV. The age shift in DHF incidence can be explained by the changes in population structure, characterized by a low birth rate. Additionally, young children attend schools equipped with air conditioning and cleaner environments, reducing their exposure to disease vectors compared to adults. Furthermore, Quang Nam province attracts a large number of workers from different regions due to its industrial zones, which contributes to changing the age structure of DHF incidence. The data suggest a higher burden of infection in the older population, which might be due to several factors, including increased exposure risk, differences in immune response, or other sociodemographic variables. However, the relatively high positivity rate in the younger age group highlights the importance of monitoring and addressing dengue infections across all age groups, including children and adolescents. In summary, the presented data emphasize the need for age-specific public health interventions and highlight that both young and older populations are at risk, with particular attention needed for understanding the dynamics affecting the younger cohort. However, due to the disparity in sample size between the two age groups, further research with a larger sample size of younger individuals would be required for a more balanced comparison. 

### 5.4. Impact of Secondary DENV Infections

The detection of IgG antibodies by ELISA in nearly half (48.06%) of the MPL RT-rPCR-positive samples indicates that a significant portion of the hospitalized DHF patients had secondary DENV infections. The presence of IgG indicates past exposure to DENV, suggesting that these cases of DHF were secondary infections rather than primary DENV infections. The reason for this conclusion is that DENV-specific IgG in the primary infections can only be detected after fourteen days of the infection [37]. Given that our samples were confirmed positive for DENV by RT-rPCR, they were collected in the early stage of DHF as the overall sensitivity of RT-rPCR is 90.64% in the first 3 days of infection [21]. The detection of IgG in these samples confirmed that these are cases of secondary DENV infections rather than primary ones. Moreover, as the blood samples involved in our study were only from hospitalized patients, this suggested that nearly half of the patients requiring hospitalization experienced DENV infection two times or more, which may lead to more severe outcomes.

Despite low-severity cases being more common, there is a noticeable increasing trend in high-severity cases (IgG-positive) over time, as shown in Figure 2. This trend is alarming and suggests that the severity of Dengue infections is escalating in the region. The increase in high-severity cases may be attributed to multiple factors, including changes in the circulating genotypes, environmental factors, and the population’s immune profile. This is critical because secondary infections are associated with more severe clinical outcomes, including Dengue hemorrhagic fever (DHF) and Dengue shock syndrome (DSS), due to the antibody-dependent enhancement (ADE) effect. The ADE effect occurs when heterotypic antibodies bind but fail to neutralize virions of the subsequent infecting DENV type, potentially enhancing viral entry into host cells and exacerbating the immune response [5,38].

Our study aligns with findings from other regions with endemic DENV transmission. For instance, a study by Tricou et al. (2011) in Southern Vietnam reported a similar pattern, with a substantial proportion of hospitalized patients showing IgG indicative of secondary infections [39]. Guzman et al. (2010) in Cuba suggested that secondary infections were associated with more severe clinical outcomes, with IgG detected in a high percentage of patients presenting with DHF [3]. The upward trend in severe infections reported in our study emphasizes the need for enhanced surveillance, better diagnostic strategies, and timely sample collection. It also highlights the importance of public health interventions such as vector control, vaccination campaigns, and public education to mitigate the impact of future DHF epidemics, particularly in regions like Quang Nam, where the risk of secondary infections remains high due to continuous DENV transmission.

### 5.5. Study Limitations

While our study provides valuable insights into DENV epidemiology in Quang Nam province from 2020 to 2022, several limitations should be noted. The absence of detailed clinical data such as symptoms, disease severity, length of hospitalization, and comorbidities, as well as demographic data including weight and height, limited our study’s ability to fully understand the progression of DHF and assess the impact of different DENV serotypes and secondary infections on disease severity. Additionally, the cross-sectional design of our study provides a snapshot of DENV prevalence and serotype distribution over the study period. However, we recognize the smaller sample size from 2020 to 2021 as a limitation, primarily due to a lower incidence of cases and logistical constraints during that period. While the larger sample size from 2022 provides robust data, the smaller 2020–2021 cohort together with the lack of longitudinal follow-up prevent us from tracking disease progression, treatment responses, and long-term immunity development among patients. Although a significant number of samples were collected, the study was limited to Quang Nam province. Expanding the findings to other regions with variations in climate, vector population, healthcare infrastructure, and population demographics would provide a more comprehensive understanding of dengue epidemiology and health burden in an endemic country like Vietnam.

### 5.6. Future Research Implications

The lower PCR positivity rate among hospitalized patients compared to the overall hospitalization rate for dengue suggests several important avenues for future research. Firstly, investigating the optimal timing for sample collection and understanding the kinetics of viral load during different stages of infection can improve PCR testing accuracy. Additionally, developing protocols for proper sample handling and storage to prevent RNA degradation is crucial. Refining clinical diagnostic criteria to differentiate dengue from other febrile illnesses can further reduce misdiagnosis rates. The presence of IgG antibodies in a significant portion of hospitalized patients indicates that secondary infections are associated with more severe clinical outcomes. Therefore, it is essential to research the mechanisms underlying antibody-dependent enhancement (ADE) to inform targeted therapies and clinical management strategies. Furthermore, the dynamic shift in DENV serotypes underscores the need for continuous surveillance to monitor changes in serotype distribution, which can help predict and prevent future outbreaks and optimize vaccine strategies. Finally, research should explore the impact of demographic factors on dengue epidemiology. Higher positivity rates in older age groups suggest varying levels of exposure and immunity, which can be better understood through longitudinal studies tracking individuals over time. By addressing these research areas, we can improve the diagnosis, treatment, and prevention of dengue, ultimately reducing its impact on affected communities.

## 6. Conclusions

Our study highlights persistent dengue prevalence and dynamic DENV serotype shifts in Quang Nam province. The lower PCR positivity rate compared to hospitalization rates underscores the need for improved diagnostic strategies and timely sample collection. Significant serotype shifts and high secondary infection rates emphasize the importance of continuous surveillance and robust public health interventions. This study reveals major shifts in the distribution of DENV serotypes, emphasizing the need for advanced surveillance for predicting and preventing further DHF epidemics. The presence of IgG in the blood samples of hospitalized patients suggests potential severe outcomes resulting from recurrent DENV infections, possibly linked to the antibody-dependent enhancement (ADE) effect. Effective surveillance, vector control, vaccination campaigns, and public education are essential for predicting and preventing future epidemics. Understanding secondary infections’ role in disease severity can enhance clinical management and therapeutic development. Continued research and investment in public health infrastructure are crucial for combating dengue in endemic regions.

## Figures and Tables

**Table 1:** Primers and probes used in the MPL RT-rPCR to detect DENV serotypes.

Serotype	Primer/Probe	Sequence
DENV-1	D1-F	CAA AAG GAA GTC GTG CAA TA
	D1-R	CTG AGT GAA TTC TCT CTA CTG AAC
	D1-PR	FAM-CAT GTG GTT GGG AGC ACG C-BHQ1
DENV-2	D2-F	CAG GTT ATG GCA CTG TCA CGA T
	D2-R	CCA TCT GCA GCA ACA CCA TCT C
	D2-PR	HEX-CTC TCC GAG AAC AGG CCT CGA CTT CAA-BHQ1
DENV-3	D3-F	GGA CTGG ACA CAC GCA CTC A
	D3-R	CAT GTC TCT ACC TTC TCG ACT TGT CT
	D3-PR	TexasRED-ACC TGG ATG TCG GCT GAA GGA GCT TG-BHQ2
DENV-4	D4-F	TTG TCC TAA TGA TGC TGG TCG
	D4-R	TCC ACC TGA GAC TCC TTC CA
	D4-PR	CY5-TTC CTA CTC CTA CGC ATC GCA TTC CG-BHQ3

**Table 2:** Overall DENV PCR positivity rates of DENV from 2020 to 2022.

	All		2020–2021		2022	
	Number of Cases	%	Number of Cases	%	Number of Cases	%
Positive	801	68.64	84	68.85	717	68.61
Negative	366	31.36	38	31.15	328	31.39
Total	1167	100	122	100	1045	100

**Table 3:** DENV positivity rates by gender.

Gender	PCR-Positive	Percentage (%)	*p*-Value
Female	414	51.69	0.121
Male	387	48.31	

**Table 4:** DENV positivity rates by age groups.

Age Group	% Among (+) Cases (*N* = 801)	Positivity Rate	*p*-Value
<16 years old (*N* = 29)	2.12 (17/801)	58.62 (17/29)	0.239
≥16 years old (*N* = 1138)	97.88 (784/801)	68.89 (784/1138)	

**Table 5:** The distribution of DENV serotypes in 2020–2022.

Serotype	All	%	2020–2021	%	2022	%	*p*-Value
DENV-1	373	46.57	5	5.95	368	51.32	<0.001
DENV-2	380	47.44	70	83.33	310	43.24	
DENV-3	0	0.00	0	0.00	0	0.00	
DENV-4	48	5.99	9	10.71	39	5.44	
Total	801	100	84	100	717	100	

## Data Availability

The datasets generated and/or analysed during the current study are available from the corresponding authors on reasonable request.
